# Public Health Emergency of International Concern (PHEIC) has Declared Twice in 2014; Polio and Ebola at the Top

**DOI:** 10.3934/publichealth.2015.2.218

**Published:** 2015-06-05

**Authors:** Mohammed A. Soghaier, Khwaja M.I. Saeed, Khushhal K. Zaman

**Affiliations:** 1 Communicable Diseases Epidemiologist & Biostatistician, Federal Ministry of Health, Khartoum, 11111, Sudan; 2 Director Surveillance/IHR Focal Point, Ministry of Public Health, Kabul, 1001, Afghanistan; 3 International Public Health/Disease Surveillance and Control Expert, Islamabad, 44000, Pakistan

**Keywords:** IHR (2005), outbreak, PHEIC, Ebola, Polio

## Abstract

**Background:**

The current Ebola outbreak in West Africa and the large scale wild Polio virus outbreak in several countries are the top most issues among international public health and scientific communities' debates and concerns. These two outbreaks were judged to be declared as Public Health Emergency of International Concern (PHEIC) during 2014. This is the first time ever to have such circumstance of two PHEICs at the same time.

**Discussion:**

PHEIC, which has to be declared by WHO Director General after a recommendation of IHR Emergency Committee; is observed to start in countries with fragile health system and conflict areas. Then it rapidly spread to threaten the global public health. The year 2014 has uniquely witnessed declaration of two events as PHEIC according to IHR (2005); Polio and Ebola Virus Disease (EVD). Both outbreaks are caused by viruses such as H1N1 which was previously declared as PHEIC in 2009.

**Summary:**

Public Health Emergencies of International Concern in 2014 occurred in countries with weak health systems and conflicts and threatening the whole globe. International collaborative work is required to contain the event and to mobilize resources/capacities between countries. Moreover, public health surveillance systems as core capacity for IHR (2005) should be strengthened in all countries with focus on those with limited capacity and ongoing conflicts. The ultimate aim is timely detection of potential PHEIC events in the future along with early preparedness and response plans.

## 1. Introduction

As per International Health Regulations (IHR-2005) [1], Public Health Emergency of International Concern (PHEIC) is defined as “an extraordinary event which is determined to constitute a public health risk to other States through the international spread of disease and to potentially require a coordinated international response” [2]. PHEIC concept is one of the major update points in IHR (2005) version. It considers prevention, protection, response and containment of an event in such a manner that is fully aligned with human right's preservation and avoidance of unnecessary interference with international travel and trade [3].

PHEIC as a requirement, has to be declared by World Health Organization (WHO) Director General (DG) after recommendation of IHR Emergency Committee (IHR EC) which is the highest specialized technical authority in this regard [4]. PHEIC has been firstly declared as a new turn in global health arena in April 2009 in the context of rapidly spreading Influenza A H1N1 pandemic [5]. During 2012–2013 there were some predications that the status of Middle East Respiratory Syndrome (Coronavirus MERS-COV) might fulfill the requirements at one point to be declared as a PHEIC, but according to IHR (2005) requirements the event was not qualifying [6].

In May 2014 Poliomyelitis was declared as PHEIC as a consequence of IHR-EC assessment (April 2014) and advice to WHO-DG. This was due to international spread of Wild Polio Virus (WPV) particularly during the known low virus transmission season (January–April/May) [7]. Then three months later in August 2014 hemorrhagic Ebola Virus Disease (EVD) in West Africa was declared as a PHEIC due to potentiality of further international spread. Ebola was particularly serious in terms of rapid communicability and disastrous outcomes [8]. Furthermore EVD had showed devastating attacks to health care providers and emergency management teams [9]. Here in this debate we are discussing the overall concept of PHEIC and the overlapping of the two events in 2014. In addition we are discussing what PHEIC means to developing countries that affected by unrest and turmoil and taking Afghanistan, Pakistan and Sudan as examples.

## 2. Discussion

The year 2014 has uniquely witnessed declaration of two PHEIC events according to IHR (2005); Polio and EVD are on the top of global public health and international scientific communities' debate and agenda. Both outbreaks are caused by viruses such as H1N1 which was previously declared as PHEIC in 2009 [10]. Both diseases have the ability for easy transmission, rapid propagation, and spread over wide geographical areas and crossing borders through different means [11]. The statistically estimated basic reproductive number (R_0_) of EVD is 2 (95% CI, 1.44 to 2.01) While it is 6 For WPV-basic reproductive number is an epidemiological parameter that indicates the average secondary cases attributed to the index case of a communicable disease [12],[13]. Both diseases have no specific antiviral medications and treatment is totally depends on symptomatic relieve, basic life and rehabilitation support interventions [14]. Not like EVD, WPV has well known and highly effective vaccines that are being used for many decades and currently the global community is implementing the Endgame strategy for eradicating the disease [15]–[17].

In 2014 WPV was actively transmitted within at least ten countries with a risk of exportation and infection to others. Eastern Mediterranean Region is shouldering the greater portion of this burden particularly due to “Pakistan to Afghanistan” and “Syria to Iraq” exportations. While, “Cameroon to Equatorial Guinea” and “Equatorial Guinea to Brazil” are the other documented exportations in the rest of world during 2014 [18]. As a response, emergency committee of IHR (2005) on polio was conducted three times and has approved the temporary recommendations for three categories of countries.

EVD in 2014 started and propagated in western Africa. Guinea was the index country which exported the infection to Sierra Leone and Liberia initially, and later to Nigeria, Senegal and Mali in addition to Spain and United States of America.

WHO had made case specific recommendations based on IHR (2005) to cope with current states of Polio and EVD. These recommendations are generally temporary and are subject to the day to day progress and developments. For Polio, recommendations in infected and exporting countries included official governmental declaration that Polio transmission is a national public health emergency. In addition to vaccination for travelers and documentation with international vaccination certificate as per IHR [19]. Regarding Ebola, recommendations for heavily infected countries included official declaration of national public health emergency, maintenance and reinforcement of high-quality exit screening and passengers tracing. Screening should involve all persons at international points of entry for unexplained febrile illness consistent with EVD infection [20].

PHEIC in developing countries that are represented in this discussion by Afghanistan, Pakistan and Sudan carries and tells several stories and lessons that need to be well taken. These countries like other several developing countries have been subject to long standing civil wars and turmoil. Two of these countries are directly involved in the current declared PHEIC related to Polio in the epidemiological block that includes both Afghanistan and Pakistan with a common viral circulation pattern and exporting the virus to each other. While Sudan is an African country that been affected by major disease outbreaks during the recent years such as Rift Valley fever outbreak in 2007 and Yellow fever outbreak in 2012. Within these countries we notice that classical index areas for spreading these outbreaks are the most vulnerable and security compromised areas. In Afghanistan, the Southern region including Kandahar and Helmand is persistently being the reservoir for WPV. The same is true for the tribal areas in northern part of Pakistan that remain as major contributor in terms of Polio related outbreaks. In Sudan, the index cases of Rift Valley fever outbreak started in the previous conflict border between Sudan and the republic of South Sudan. In 2012, the yellow fever outbreak started and spread from security compromised areas in newly formed central Darfur state in the western part of the country.

Eastern Mediterranean Region Office for WHO that including theses three countries had recently conducted an assessment for country preparedness to EVD potential case importations. It revealed that countries have very low capacity to respond and control potential outbreak despite the fact that technical guidance have been provided to these countries. It is now clear more than any given time before that health systems should be adequately built in all such countries. National governments of these countries are required to prove the commitment to implementation of IHR (2005). Availing adequate or even the minimally required capacities to cope with any PHEIC in a country is the major determinant of the final outcome. This also could be concluded from the recent experience of response to current PHEIC. Despite the huge efforts that paid by international community represented by the United Nation Mission for Ebola Emergency Response [16],[21] and partners, the overall outcome and timeliness of emergency control was not satisfying. Several observers criticized the way of operation, technical response and leadership at the field level [8]. In the same regards repeated extensions of targeted deadline for achieving global Polio eradication is another critique point against the seriousness and effectiveness of international /global initiatives to control PHEICs [22],[23].

## 3. Conclusion

In this brief discussion, it is clear that Public Health Emergencies of International Concern occurs in countries with weak health systems and internal conflicts then threatening the whole globe. International collaborative work is required to contain the event and mobilize resources/capacities between countries. Public health surveillance systems as core capacity for IHR (2005) should be strengthened globally aiming for timely detection of potential PHEIC events in the future. Building capacities for implementation of IHR in war/turmoil stricken countries with low resources, weak and fragile health systems will guarantee early detection, response and control and PHEIC in other parts of the world.

## Abbreviations

PHEIC: Public Health Emergency of International Concern
WHO: World health Organization
IHR: International Health Regulations
EC: Emergency Committee
EVD: Ebola Virus Disease
MERS: Meddle East Respiratory Syndrome
COV: Coronavirus
DG: Director General
WPV: Wild Polio Virus
R_0_: Basic Reproductive Number
